# Prevalence of Non-communicable Disease Risk Factors Among Adults in a Rural Field Practice Area of a Tertiary Care Medical Center in Karnataka

**DOI:** 10.7759/cureus.67474

**Published:** 2024-08-22

**Authors:** Sankiya M, B N Sunil, Varun R

**Affiliations:** 1 Community Medicine, Sri Devaraj Urs Medical College (SDUMC) Sri Devaraj Urs Academy of Higher Education and Research (SDUAHER), Kolar, IND

**Keywords:** cbac, occupational exposure, cancer, family history, physical inactivity, rural area, waist circumference, hypertension, diabetes mellitus, risk factors of ncds

## Abstract

Background

Non-communicable diseases (NCDs), also referred to as chronic diseases, typically have a long duration and arise from a combination of genetic, physiological, environmental, and behavioral factors. Each year, 17 million people under the age of 70 die from non-communicable diseases (NCDs), with 86% of these premature deaths occurring in low- and middle-income countries.

Objectives

To estimate the prevalence of NCD risk factors among adults (18-65 years) in a rural population.

Methods

A cross-sectional study was conducted by selecting 200 participants from 200 households using convenience sampling. Participants aged 18-65 years were included, and locked households were excluded. Sociodemographic profiles were assessed using semi-structured questionnaires, and NCD risk factors were assessed using a Community-Based Assessment Checklist (CBAC). Descriptive statistics and associations were analyzed.

Results

The majority of participants were men (53.5%), married (89.5%), and belonged to the class 2 socioeconomic classification. The prevalence of NCD risk factors was 17%, with smoking (12.5%), alcohol consumption (6%), and waist circumference (1.8% for men and 27.9% for women) being the most common risk factors. Older age, lower educational attainment, unemployment, and lower-income classes were associated with a higher risk of NCDs.

Conclusion

The study identifies key risk factors for non-communicable diseases (NCDs) as family history, waist circumference over 90 cm, daily alcohol consumption, and tobacco use, all significantly increasing the risk. Physical activity under 150 minutes per week and occupational exposure to crop residue showed no significant effect.

## Introduction

A combination of genetic, physiological, environmental, and behavioral factors typically cause non-communicable diseases (NCDs), also known as chronic diseases, which typically have a long duration. Every year, 17 million people under the age of 70 die from non-communicable diseases (NCDs), with 86% of these premature deaths occurring in low- and middle-income countries [1]. Premature mortality from NCDs is measured as the unconditional probability of dying between the ages of 30 and 70 from any of the four major non-communicable diseases: cardiovascular diseases, cancers, diabetes, and chronic respiratory diseases [2]. The goal of the Global NCDs Compact 2020-2030 is to accelerate progress in NCDs prevention and control. It seeks to ensure that member states implement programs and policies that improve NCD outcomes and extend the lives of individuals who are affected by them [3].

In India, the primary causes of morbidity and mortality have traditionally included maternal and child ailments, as well as communicable infections. However, injuries and chronic illnesses such as cancer, diabetes, mental health conditions, and cardiovascular diseases are also significant causes of death and disability [4]. NCDs have a variety of causes, including underlying, modifiable, non-modifiable, and intermediate risk factors. Research reveals that socioeconomic, cultural, political, and environmental factors, including population aging, globalization, urbanization, and nutrition change, all contribute to an increase in NCDs in emerging countries. [5]. Over the next 25 years, significant increases in the percentage of the disease burden attributed to these conditions are predicted. Chronic illnesses are common in both rural and low-income populations. There are many affordable ways to prevent these illnesses, such as a healthy diet, regular exercise, vaccination, routine health screening, smoking cessation, etc., at the primary and secondary levels, but people in rural and low-income communities rarely use them. This is due to a lack of awareness, limited access to healthcare, and other socio-economic challenges [6].

Although these illnesses are frequently linked to older age groups, data indicates that 17 million deaths from NCDs happen before the age of 70. It is estimated that 86% of these premature deaths take place in low- and middle-income nations [1]. The primary risk factors for NCDs, including sociodemographic factors, tobacco use, alcohol abuse, and physical inactivity, often worsen early in life due to lifestyle choices, making the majority of these diseases preventable [7]. The primary objective of this study is to estimate the prevalence of risk factors for non-communicable diseases (NCDs) among individuals aged 18 to 65 in the rural community of Devarayasamudra, Kolar District, Karnataka, India.

## Materials and methods

Study design and settings

A cross-sectional method was used for this study, which was done by visiting the houses of rural regions of Devarayasamudra in the Kolar district of Karnataka, India, using the interview method. The study was conducted at Sri Devaraj Urs Medical College, located in Kolar, Karnataka.

Sample size calculation

n=Za^2 ^(p)(q)/d^2^

n=sample size; Zα^2^=power of the study at 95% confidence interval=1.96; P=prevalence=50% [8]; q=1-p; d=absolute error=7

n=(1.96)^2^x50x50/7^2^=196

n=196

Study population

A total of 200 participants were selected from the village of Devarayasamudra. Since data collection took place during the day in a rural area, many adults, primarily farmers and daily laborers, were away at work and unavailable for participation. To address this, a convenient sampling method was used. The concept of a household is based on the arrangements made by individuals or groups to provide themselves with food and other essentials for living [9]. The adult household members (aged 18 to 65 years) of 200 selected houses were interviewed. The houses that were locked despite two visits were excluded from the study, and informed consent was obtained from the participants.

To assess the sociodemographic profile of the participants, semi-structured questionnaires were utilized. The Community Based Assessment Checklist (CBAC) was used to identify risk factors for non-communicable diseases (NCDs) [10]. The checklist includes factors such as engaging in less than 150 minutes of physical activity per week, daily alcohol consumption, being over 50 years of age, daily smoking, family history of NCDs, and waist circumference measurements exceeding 100 cm for men and 90 cm for women. Each of these factors contributes to a total risk score, with a score of more than four indicating an increased risk of developing NCDs. Additionally, "yes" or "no" responses were used to assess other risk factors, including those related to oral and breast cancer, as well as occupational exposures. This comprehensive assessment provides a clear understanding of the participants' risk profiles for NCDs.

Data analysis

The collected data were cleansed, coded, entered in Microsoft Excel (Microsoft Corporation, Redmond, Washington, United States), and exported to IBM SPSS Statistics for Windows, Version 22 (Released 2013; IBM Corp., Armonk, New York, United States) for analysis.

Variables

NCD risk factors were the dependent variables in this study. The independent variables included factors such as age, gender, caste, education, occupation, marital status, family type, income, personal and family history, waist circumference, alcohol and smoking consumption, physical activity, and occupational exposure.

To summarize the dependent variable and independent variables, descriptive statistics were conducted. To estimate the association of various factors with NCDs, an odds ratio was calculated and a 95% confidence interval was determined. Using the chi-square test, the association of risk factors for NCDs was identified. The odds ratio (OR) in our analysis was obtained by comparing the odds of being at high risk for non-communicable diseases (NCDs) among participants exposed to specific sociodemographic factors or behaviors with those who were not exposed. We categorized participants into "high risk" and "no risk" groups based on their assessment scores. The OR was calculated using the ratio of the odds of high risk in the exposed group to the odds in the non-exposed group. Confidence intervals (CI) were also determined to assess the precision of the OR, and statistical significance was evaluated using p-values, and <0.05 was considered statistically significant.

Ethical clearance

Institutional ethical committee approval was obtained (No. SDUMC/KLR/IEC/327/2023-2024).

## Results

Prevalence of NCDs

The study utilized the CBAC, which revealed that 34 out of 200 participants had the score higher than four, indicating they are at high risk for developing non-communicable diseases (NCDs), resulting in a prevalence rate of 17% among study subjects.

Sociodemographic detail

Table 1 detailed the sociodemographic factors affecting non-communicable disease risk among 200 individuals. Significant factors include age, with those under 50 having a lower risk than those over 50 (OR: 13.098, p<0.001); caste, with the general caste at lower risk than other backward castes (OR: 4.913, p<0.020); education, with literates at lower risk than illiterates (OR: 7.901, p<0.001); and marital status, with singles at lower risk than married individuals (OR: 11.681, p<0.003). Gender, employment status, family type, income, and personal disease history do not significantly influence NCD risk.

**Table 1:** : Association between socio-demographic details and risk of non-communicable diseases (n=200) * statistically significant (P-value<0.05) DM: diabetes mellitus; HTN: hypertension

Sl. No	Socio-demographic details	Total (%) (N=200)	Non-communicable disease		Odd’sratio (Confidence interval)	P-value
			No risk	High risk		
1.	Gender					
	Males	107 (53.5%)	72 (67.3%)	35 (32.7%)	1.079 (0.599-1.944)	0.800
	Females	93 (46.5%)	61 (65.6%)	32 (34.4%)		
2.	Age					
	<50 years	141 (70.5%)	133 (94.3%)	8 (5.7%)	13.098 (5.437-31.558)	0.001*
	>50 years	59 (29.5%)	33 (56%)	26 (44%)		
3.	Caste					
	General	41 (20.5%)	39 (95.1%)	2 (4.9%)	4.913 (1.126-21.432)	0.020*
	Other backward caste	159 (79.5%)	127 (79.9%)	32 (20.1%)		
4.	Education					
	literate	139 (69.5%)	112 (80.6%)	27 (19.4%)	7.901 (4.023-15.517)	0.001*
	Illiterate	61 (30.5%)	21 (34.4%)	40 (65.6%)		
5.	Occupation					
	Employed	145 (72.5%)	118 (81.4%)	27 (18.6%)	0.637 (0.260-1.562)	0.322
	Unemployed	55 (27.5%)	48 (87.3%)	7 (12.7%)		
6.	Marital status					
	Single	21 (10.5%)	20 (95.2%)	1 (4.7%)	11.681 (1.532-89.049)	0.003*
	Married	179 (89.5%)	113 (63.1%)	66 (36.8%)		
7.	Type of family					
	Joint	105 (52.5%)	86 (82%)	19 (18%)	0.849 (0.404-1.783)	0.665
	Nuclear	95 (47.5%)	80 (84.2%)	15 (15.8%)		
8.	Income					
	<5000	154 (77%)	125 (81.2%)	29 (18.8%)	0.526 (0.191-1.447)	0.207
	>5000	46 (23%)	41 (89.1%)	5 (10.9%)		
9.	Personal history					
	Present (DM, HTN, Others)	105 (52.5%)	86 (82%)	19 (18%)	0.849 (0.404-1.783)	0.665
	Absent	95 (47.5%)	80 (84.2%)	15 (15.8%)		

NCD risk factors and non-communicable disease

Table 2 examines risk factors for non-communicable diseases (NCDS) among 200 individuals. Significant predictors include family history, with 71.4% of those having a family history (17.5% of the sample) at high risk (OR: 7.321, p<0.001); waist circumference over 90 cm (36.5%) (OR: 2.270, p<0.029); daily alcohol consumption (7.5%) with 80% at high risk (OR: 9.455, p=0.000); and tobacco use, with 51.6% of users (15.5%) at high risk (OR: 8.948, p<0.001). Physical activity under 150 minutes per week (58%) shows a non-significant trend towards higher risk (OR: 0.617, p=0.119), and occupational exposure to crop residue shows no significant effect (OR: 1.451, p=0.349). Key risk factors for NCDs are family history, waist circumference, alcohol consumption, and tobacco use.

**Table 2:** Association between NCD risk factors and risk of non-communicable disease * statistically significant (P value <0.05) Other activities include burning garbage leaves and working in industries that expose workers to smoke, gas, and dust, such as brick kilns and glass factories.

Sl.No	Risk factors	Total (%) (N=200)	Non-communicable disease			Odd’s ratio (Confidence interval)	P-value
			No risk		High risk		
1	Family history						
	Yes	35 (17.5%)	10 (28.6%)	25 (71.4%)		7.321 (3.248-16.503)	0.001*
	No	165 (82.5%)	123 (71.5%)	42 (25.5%)			
2	Waist circumference						
	80cm/less	127 (63.5%)	111 (87.4%)	16 (12.6%)		2.270 (1.076-4.792)	0.029*
	More than 90 cm	73 (36.5%)	55 (75.3%)	18 (24.7%)			
3	Do you consume alcohol daily?						
	Yes	15 (7.5%)	3 (20%)	12 (80%)		9.455 (2.567-34.825)	0.001*
	No	185 (92.5%)	130 (70.3%)	55 (29.7%)			
4	Do you smoke or consume smokeless products such as gutka or khaini?						
	Never	169 (84.5%)	151 (89.3%)	18 (10.7%)		8.948 (3.797-21.090)	0.001*
	Past/Daily	31 (15.5%)	15 (48.4%)	16 (51.6%)			
5	Do you undertake any physical activities for minimum of 150 minutes in a week?						
	At least 150 minutes in a week	84 (42%)	61 (72.6%)	23 (27.4%)		0.617 (0.336-1.134)	0.119
	Less than 150 minutes in a week	116 (58%)	72 (62%)	44 (38%)			
6	Occupational exposure						
	Crop residue	79 (39.5%)	68 (86%)	11 (14%)		1.451 (0.664-3.172)	0.349
	Others	121 (60.5%)	98 (81%)	23 (19%)			

## Discussion

This community-based cross-sectional study involving 200 participants from the rural village of Devarayasamudra sought to assess the prevalence of non-communicable disease (NCD) risk factors among adults and to explore the associations between these risks and various demographic and lifestyle factors. Older age, caste, education level, marital status, family history, larger waist size, alcohol use, and smoking were all strongly connected to a higher risk of NCDs. On the other hand, although there were certain significant changes, gender, work status, income, family structure, and physical activity did not strongly correlate with the risk of NCDs. These results suggest that public health efforts in rural areas should focus on both social and lifestyle factors to reduce NCD risks.

Age emerges as a potent determinant, with older individuals, particularly those over 50 years of age, exhibiting a markedly higher risk than younger age groups. The current study shows individuals aged 18 to 65, with a particular emphasis on those aged 45-64, as this group is experiencing an early onset of chronic diseases, contributing to the risk of premature death from non-communicable diseases (NCDs). The cutoff point of 50 years was used to differentiate between younger and older adults, aligning with epidemiological practices and highlighting the increased risk of NCDs that typically emerges after this age. This age range is crucial because it includes a significant portion of the working population, and the early development of conditions like heart disease, stroke, and diabetes in this group poses serious public health and economic challenges. Addressing these risks through targeted interventions is vital to reducing premature deaths and mitigating future healthcare burdens [11].

According to the study by Syed et al. (2023), the prevalence of NCDs tends to increase with age, with the highest increases noted among individuals aged 30 to 49 years. Additionally, educational attainment is a crucial factor, with literate individuals demonstrating a lower risk compared to their illiterate counterparts, which is similar to the current study [12]. A survey conducted between 2005 and 2016 among middle-aged individuals in Japan found a correlation between lower educational attainment and a higher incidence of diabetes and stroke, both of which are diseases that increase the risk of non-communicable diseases (NCDs) in both men and women. Additionally, women with lower educational levels were found to have a higher prevalence of hypertension, another condition that contributes to the risk of NCDs [13,14].

Occupation also plays a significant role, with professional workers displaying the lowest risk and unemployed individuals showing the highest risk. However, the current study shows that employment is not significant because more people are employed. Moreover, marital status and family structure exhibited notable associations, with married individuals and those from nuclear families displaying higher risks of NCDs, which is similar to the current study [15].

Higher income classes generally exhibit lower health risks, reflecting significant disparities in access to healthcare, education, and resources. These inequalities are prominent in urban areas like Mumbai and Delhi, where socioeconomic status plays a crucial role. However, when comparing with the current study conducted in a rural area, it is evident that rural regions face even greater challenges, as limited infrastructure and fewer resources exacerbate health disparities. Similar patterns are seen in countries like the United States and Sweden, where social hierarchies impact health outcomes, but the rural-urban divide in India intensifies these effects [15].

Our study examined the association between family history and the risk of developing non-communicable diseases (NCDs). We found that individuals with a family history of NCDs are significantly more likely to develop these conditions, with an odds ratio of 7.321 (95% CI: 3.248-16.503) and a highly significant p-value of 0.00. This aligns with findings from a cross-sectional study in the United States, which highlighted that a family history of diabetes not only increases the risk but also serves as a valuable tool for early detection and prevention. Individuals with a moderate to high familial risk were more likely to be diagnosed with the disease [16].

Another study conducted by Kuruvilla et al. highlighted that non-modifiable risk factors, such as family history and age, play a significant role in the development of non-communicable diseases (NCDs). According to their findings, individuals with a family history of chronic diseases were 2.17 times more likely to develop NCDs, whereas those over the age of 50 had a 3.47 times higher risk, which is similar to the current study. Additionally, modifiable risk factors such as obesity (odds ratio, 8.34), unhealthy waist-to-hip ratio, and hypertension (odds ratio, 1.85) have been identified as contributors to increased susceptibility to NCDs. In our current study, while we observed waist circumference, body mass index (BMI) and waist hip ratio were not measured. However, we did assess chronic diseases, although they were not found to be statistically significant [17].

In contrast, a study by Nawoda Hewage et al. found a statistically significant relationship between waist circumference and NCD risk in both men and women (p=0.029*). Similar to our findings, this study suggests that an increased waist circumference raises the likelihood of developing NCDs [18].

In a study by Vivek K. Mishra et al., it was found that women who smoked, used smokeless tobacco, or consumed alcohol had a significantly higher risk of developing NCDs (16%, 8%, and 20% higher, respectively) compared to women who did not engage in these behaviors. The population-attributable risk was 1.8% for smoking, 0.8% for smokeless tobacco use, and 2.2% for alcohol consumption, all with a p-value of less than 0.001. In our current study, no women reported smoking or drinking alcohol, but the findings suggest that these behaviors greatly increase the risk of NCDs [19].

A study by Nalinee Jakkaew et al. found that among men, a higher risk of harm from alcohol use was associated with five out of eight NCD risk factors: smoking, physical inactivity, higher blood pressure, higher blood glucose, and higher triglyceride levels. In our current study, alcohol consumption and smoking were significantly associated with NCD risk factors, while physical inactivity did not show a significant impact. This lack of significance may be due to the fact that our study population primarily consisted of farmers, who typically engage in physically demanding work, naturally reducing the risk associated with inactivity. Additionally, cultural and social practices in rural areas often involve more movement, and dietary habits may include more whole foods, which could mitigate the health risks of physical inactivity. It's also possible that underreporting or misclassification of physical activity occurred, as participants may not recognize their daily activities as exercise [20].

A study in China by Xiang Huang et al. found that over one-third of the elderly population suffered from major non-communicable chronic diseases (NCDs) like cardiovascular diseases, diabetes, and chronic respiratory diseases. The study highlighted that those who engaged in physical activity or could climb three flights of stairs were less likely to develop NCDs, while those with difficulty bending, kneeling, or squatting were more at risk, likely due to the physical demands of these activities. In our study, although 150 minutes of weekly physical activity was associated with a lower NCD risk, the correlation was not statistically significant. However, promoting physical activity remains crucial for overall health, especially in rural settings where high levels of physical labor and active lifestyles, along with healthier dietary habits, may reduce the impact of inactivity on NCD risk [21].

In a 2024 study by Susan Peters et al., the narrative review underscores specific exposures associated with non-communicable diseases (NCDs), particularly highlighting diesel engine exhaust and cadmium as significant risk factors linked to lung cancer. On comparison with the current study, participants engage in multiple types of work, leading to mixed or intermittent exposures that are harder to quantify accurately. Also, variability in environmental conditions, such as seasonal changes and differing agricultural practices, can also complicate the assessment of occupational risks. Moreover, limited access to healthcare in rural areas might contribute to underdiagnosis or misreporting of NCDs [22].

Limitations 

This study, which examined socio-demographic and risk factors for non-communicable diseases (NCDs), has few limitations. The sample size of 200 participants is relatively small, which may limit the generalizability of the findings. The cross-sectional design captures data at a single point in time, making it difficult to establish a causal relationship between risk factors and NCD development. The scope of risk factors examined is limited, potentially overlooking other relevant factors like diet, stress, and mental health. Also in rural communities, the risk factors for non-communicable diseases (NCDs) are closely tied to the unique social and epidemiological conditions of the area. Additionally, the predominance of agricultural work exposes individuals to occupational hazards and influences dietary habits, both of which are crucial in understanding NCD risks. Limited access to healthcare, lower education levels, and traditional lifestyles further complicate the public health challenges. To effectively address NCDs in these settings, it is essential to study these interconnected factors and design interventions tailored to the specific needs of rural populations.

## Conclusions

In conclusion, the data from the current study highlights the critical risk factors that contribute to the development of non-communicable diseases (NCDs) within the studied population. Family history, alcohol consumption, age, education, and tobacco use have emerged as significant determinants of the prevalence of NCDs. Understanding and addressing these risk factors through targeted interventions and public health initiatives are essential for mitigating the burden of NCDs and promoting overall well-being.
